# Multiple Mini Interview Performance and Career Maturity related to Medicine

**DOI:** 10.15694/mep.2018.0000284.1

**Published:** 2018-12-13

**Authors:** Ralph Manuel, Nikki Zaidi, Ryan Duffy, Nicole Borges

**Affiliations:** 1 University Of Mississippi Medical Center; 2 University of Michigan School of Medicine; 3 University of Florida; 4 University of Mississippi Medical Center

**Keywords:** Career maturity, Medical Students, Multiple Mini Interview

## Abstract

This article was migrated. The article was marked as recommended.

**Introduction:** Multiple Mini Interview (MMI) is used to determine the most suitable candidates for the medical profession. It is also important to ensure students have the career maturity necessary to cope with the developmental tasks associated with medical training. This study explored if students stronger in their career decision of medicine perform better on the MMI.

**Methods:** 119 students (72% response rate) completed a career maturity measure. Student MMI scores were matched to career maturity responses.

**Results:** A significant positive correlation (p <.05) existed between MMI score and specify physician as a career preference (r =.177, *N* =112, p <.031). There was no significant correlation with the MMI score and crystalize a career preference (r =.046, *N* =119, *p* =.31) or implement physician as career choice (r =.039, *N* =114, *p* =.34). Mean scores for both crystalize career preference ( *M* = 18.91) and implement physician as career choice ( *M* = 17.46) indicate that students performing lower on the MMI continue coping with those tasks as compared to specify (M=20.65).

**Conclusion:** Findings suggest a relation between medical student’s career maturity and interview performance as measured using the MMI, with students in the intermediate stages of career development having higher MMI scores.

## Introduction

Medical schools endeavor to admit applicants most suitable for a career in medicine. The processes used to achieve this goal involve a range of selection factors meant to elicit both cognitive and non-cognitive applicant traits. While selection criteria continue to evolve ( Wilson, Roberts, Flynn, & Griffin, 2012), the medical school preadmission interview (MSPI) remains one of the most widely used tools in medical school admissions ( Monroe, Quinn, Samuelson, Dunleavy & Dowd, 2013). Over the past decade, the multiple mini-interview (MMI) ( Eva, Rosenfeld, Reiter, & Norman, 2004) has gained considerable attention as an alternative to more traditional MSPI formats. The MMI typically uses scenarios that evoke the type of decision-making skills necessary to effectively negotiate challenges faced in patient care and the practice of medicine. The MMI is a multi-sampling, structured interview format in which raters assess applicants using several 5-15 minutes interview stations, each with a different rater and interview scenario ( Eva, Rosenfeld, et al., 2004; Pau et al., 2013). Each MMI station is assigned a specific scenario that focuses on domains such as “knowledge of the healthcare system” and “communication skills,” which are considered necessary for a career in medicine ( Eva, Rosenfeld, et al., 2004).

The MMI has been lauded for increasing the low reliability estimates that plague traditional MSPIs, and the extant literature reports moderate to high generalizability for medical school MMIs ranging 0.58-0.81 ( Eva, Reiter, Rosenfeld, & Norman, 2004a; Eva, Reiter, Rosenfeld, & Norman, 2004b; Eva, Rosenfeld et al., 2004; Uijtdehaage, Doyle, & Parker, 2011, Dowell et. al, 2012). In addition, studies suggest that MMI scores can be used to predict performance on medical school clerkships and on medical licensure examinations ( Eva et al., 2004a; Eva et al., 2009; Eva et al., 2012; Husbands & Dowell, 2012). Although current studies examine predictive validity as evidenced by academic success, these traditional criterion variables may not fully demonstrate suitability for medicine but instead demonstrate ability to perform well under standardized testing conditions ( Lubelski, 2013). Consequently, there is a growing need to identify alternative criterion variables that better align with the MMI.

In light of growing evidence that medical school performance is linked with personality ( Doherty & Nugent, 2011), the association between personality and MMI scores has been examined. Initial studies, however, report conflicting evidence regarding this potential association. Using the NEO-5 Personality Inventory, Kulasegaram et al. (2010) found no statistically significant correlations between any of the personality factors and the MMI. Conversely, Griffin and Wilson (2012) used another scale, the International Personality Item Pool, to measure personality and found the MMI to be significantly correlated with three of the five factors of personality-extraversion, conscientiousness, and agreeableness; and similarly, Jerant et al. (2012) used the Big Five Inventory and found through regression analysis that applicants with higher extraversion scores had significantly higher MMI scores (adjusted parameter estimate = 5.93 higher, 95% CI: 4.27-7.59; p < .01). Emotional intelligence has also been explored as a possible proxy measure for the MMI, although no significant association was found when using the Bar-On EQ-i emotional intelligence (EI) instrument ( Yen, Hovey, Hodwitz, & Zhang, 2011).

Just as medical schools strive to elicit information about applicants through the MMI in an attempt to determine the most suitable candidates, medical schools also want to ensure that their students have the career maturity necessary to cope with the developmental tasks associated with medical training. Career maturity reflects an individual’s ability to make sound choices about one’s career. The developmental tasks related to physician career maturity are described by Savickas (1984) with the first three tasks associated with the “career cycle.” These tasks include “career crystallization” in which a general preference for a career in medicine is formulated along with the formation of a vocational identity; followed by “career specification” in which a general preference is transformed into a specific preference for a career in medicine; and “career implementation” in which these preferences compel action driven by goal-directed behaviors. The next three tasks are associated with the “occupational cycle.” These tasks include the crystallization of a specialty choice, the “occupational crystallization,” which involves the formulation of a generalized preference for an occupational role that aligns with those in a particular physician specialty; “occupational specification” which requires the person to convert the generalized occupational preference into a specific specialty preference; and “occupational implementation,” in which the specialty preference is converted into a fact by occupying a residency position. Finally, the last task associated with physician career maturity involves “stabilization” in terms of settling down and establishing a practice position for a period of time ( Savickas, 1984).

Studies suggest that career maturity, as captured by the Medical Career Development Inventory (MCDI), increases with increased exposure to medicine. Borges et al. (2007) found a significant difference in career maturity between first-year medical students matriculating from a traditional academic program versus students from an accelerated program. Students from an accelerated program were found to have lower career maturity when compared with students from a traditional undergraduate program. This difference is largely attributed to the truncated undergraduate educational experience associated with the accelerated program that does not provide students adequate time to increase their level of career maturity and readiness for career decision-making. Similarly, Duffy et al. (2011) found that medical students’ vocational development increased from their first year of medical school to the third year (MCDI total score = 99.41 vs. 109.11, p < .01). In light of the fact that career maturity is a developmental process that increases over time and exposure to medicine, MCDI scores may serve as a criterion variable for assessing suitability for medicine; however, previous research has not examined the potential association between career maturity and preadmissions factors. An association between the MMI and the MCDI could provide further validity evidence to support the continued use of the MMI in medical school admissions. Specifically, this study examined whether students with greater career maturity in their decision to pursue medicine perform better on the MMI.

## Methods

This study was granted exempt status by the study site’s institutional review board. All students selected into the entering class of 2009 were required to participate in an MMI as part of the preadmissions review process. Participation in this study, however, was based on written consent and voluntary completion of the MCDI after matriculation. Following completion of the MCDI, student MMI scores were matched to their MCDI responses. Only MCDI scores pertinent to the dimensions of career maturity associated with the career cycle- career crystallization, career specification, and career implementation were used for purposes of this current study.

The MCDI is a tool used to assess developmental tasks related to physician career maturity ( Savickas, 1984), and this tool has produced reliable scores with estimates ranging 0.73 to 0.94 ( Duffy et al., 2011; Henry et al., 1992; Savickas, 1984). The MCDI consists of a 35-item inventory that uses a modified 5 point Likert-scale to assess seven tasks associated with a dimension of career maturity to measure thinking and planning activities that facilitate vocational development.

We examined associations between students’ MMI and MCDI scores using the Pearson Correlation Coefficient R. We used *p* <.05 as the standard for statistical significance. To perform statistical analyses, we used IBM SPSS Statistics.

## Results/Analysis

MMI and MCDI scores from 119 students who matriculated to one United States medical school in 2009 were analyzed. Overall, participation was high with a 72% response rate. A small but significant positive correlation was found between MMI scores and the dimension of career maturity related to specifying physician as a career preference (r =.177, *N* =112, *p* <.031). There was no significant correlation with the MMI score and the other dimensions of career maturity; crystalizing a career preference (r =.046, *N* =119, *p* =.31) or implementing physician as career choice (r =.039, *N* =114, *p* =.34). Mean scores for both crystalize a career preference ( *M* =18.91) and implement physician as career choice ( *M* =17.46) indicate that students performing lower on the MMI continue coping with those tasks. Specify career (M=20.67) indicates that students have successfully completed the tasks associated with this dimension ( Savickas, 1984).

Further analysis by gender indicated there was a significant correlation for male MMI scores and implement physician as a career choice (r =.289, *N* = 60, *p* =.025); there were no other significant correlations by gender.

## Discussion

Our findings suggest there is a relation between medical students’ career maturity and performance during their medical school interviews as measured using the MMI with students in the intermediate stages of physician career maturity having higher MMI scores. This indicates that there is an association between MMI scores and medical students who are more self-assured in their desire to become a physician and who have taken steps to self-assess their suitability and viability for becoming a physician (i.e. career specification). This is important not only in making the commitment to medical school, but in considering students’ career development and the ability to self-assess as it pertains to future career choices. As Cruess et al. (2015) describe; “who they are” at the beginning and “who they wish to become” is dynamic and part of professional identity formation (p. 718).

This study is constrained by some limitations. First, this study involved only one year of data from one institution. Therefore, caution should be used in generalizing the results. Although the results of this study demonstrate a degree of validity evidence, further analysis is warranted to account for additional covariates that could influence both MMI and MCDI scores.

## Conclusion

This career maturity and ability to self-assess may manifest itself during the preadmissions selection process in terms of MMI scores that reflect students’ self-assurance and self-confidence in addressing scenarios. Further research could include continued exploration of factors influencing career maturity, such as the amount of specific exposure to medicine (e.g. shadowing) and its relation to the MMI. If such a relation exists, students who have more access to shadowing could have an advantage over groups of students that do not have similar opportunities and access; and perhaps this could help explain why some disadvantaged students do not do well on the MMI ( Jerant et al., 2015). Students who have a better understanding of themselves from a career development standpoint might also be able to better navigate their medical education, including decisions choosing a residency that matches their strengths and abilities and overall future career planning.

## Take Home Messages

Our findings suggest there is a relation between medical students’ career maturity and performance during their medical school interviews as measured using the MMI with students in the intermediate stages of physician career maturity having higher MMI scores.

## Notes On Contributors

R. Stephen Manuel, PhD is Associate Dean of Admissions at the University of Mississippi Medical School, Jackson, Mississippi, USA.

Nikki Zaidi, PhD is Associate Director, Advancing Scholarship, Office of Medical Student Education, University of Michigan Medical School, Ann Arbor, Michigan, USA.

Ryan D. Duffy, PhD is Professor in the Psychology Department at the University of Florida, Gainesville, Florida, USA.

Nicole J. Borges, PhD, is Professor in the Department of Neurobiology and Anatomical Sciences, and Chief Education Officer, Research and Scholarship, Office of Mississippi Physician Workforce, University of Mississippi Medical Center, Jackson, Mississippi, USA.
