# Supplementary material for: Multiple Mini Interview Performance and Career Maturity related to Medicine
Source: MedEdPublish (2016). 2018 Dec 13;7:284. [Version 1] doi: 10.15694/mep.2018.0000284.1 (PMC10711951; doi:10.15694/mep.2018.0000284.1)
Supplement: Peer review history

# Reviewer report: Reviewer response for version 1

**DOI:** 10.21956/mep.19732.r29560

**Published:** 2019-04-16

**Authors:** Trevor Gibbs

**Affiliations:** 1 AMEE

This review has been migrated. The reviewer awarded 4 stars out of 5 Despite being able to almost predict the results of this study from its title, I did enjoy reading this paper and indeed learned from it. I do think that it asks more questions than answers and particularly in the area of opening access to medical school admissions and how we deal with students from disadvantaged backgrounds. A good read for those involved in student selection.

Reviewer Expertise:

NA

No decision status is available

# Reviewer report: Reviewer response for version 1

**DOI:** 10.21956/mep.19732.r29559

**Published:** 2018-12-26

**Authors:** John Cookson

**Affiliations:** 1 University of Worcester

This review has been migrated. The reviewer awarded 4 stars out of 5 This is a useful addition to the literature on MMI. Perhaps not suprisingly increased career maturity was related to higher marks in the MMI, but it does suggest that the MMI is measuring something real in which we are interested. The problem is knowing what to do with the information. As the authors discuss, and as the overview of the series considers, we need a clearer view of what we are looking for before we can use this sort of information. If we want to widen participation are we prepared to accept those who have less of a defined view about their career because they have had fewer opportunities in their lives to refine that view?

Reviewer Expertise:

NA

No decision status is available
